# Mental health and substance misuse 7 years following an Emergency Department admission for alcohol intoxication

**DOI:** 10.1186/1940-0640-10-S2-P3

**Published:** 2015-09-24

**Authors:** A Adam, M Faouzi, B Yersin, P Bodenmann, JB Daeppen, N Bertholet

**Affiliations:** 1 Lausanne University Hospital, Lausanne, Switzerland; 2 Department of Community Medicine, University of Lausanne, Lausanne, Switzerland

## Background

How young adults evolve at a distance of being admitted for alcohol intoxication in the Emergency Department (ED) is not well characterized.

## Objective

Assess the prevalence of alcohol use disorder (AUD), substance use and health status 7 years following an ED admission for alcohol intoxication.

## Methods

In 2006-2007, 631 patients aged 18-30 were admitted for alcohol intoxication at the ED of a tertiary Swiss hospital. In 2014, they were re-contacted and interviewed to complete: demographics, alcohol use disorders identification test-consumption (AUDIT-C), Mini International Neuropsychiatric interview (MINI) for AUD, SF12 mental and physical component summary scores (MCS, PCS), Patient Health Questionnaire (depression and anxiety disorders), past year use of illegal drugs/tobacco, if they remembered the admission and discussing their drinking while admitted.

## Results

In 2014, 318/631(50.4%) patients completed the questionnaire: 32.1% were women, 36.5% unemployed, 73.6% remembered the admission and 34.6% discussing their drinking; 65.1% had AUDIT-C≥4 (i.e. positive screen for AUD). According to the MINI, 15.1% had alcohol dependence and 13.2% harmful use. 18.6% had depression, and 15.4% an anxiety disorder. Mean (SD) PCS and MCS were 52.2(9.3) and 42.7(11.7). Prevalence of any use (past year) was 80.2% for tobacco, 53.1% for cannabis, 22.6% for cocaine, 13.5% for sedatives, 11.0% for stimulants, 7.2% for opioids, and 6.0% for hallucinogens. At least once a week use was 65.4% for tobacco, 25.5 % for cannabis, 3.8% for cocaine, 7.2% for sedatives, 0.9% for stimulants 3.8% for opioids and 0% for hallucinogens. No differences were found between those who completed the questionnaire and those who did not on 2006-2007 alcohol intoxication admission data (age, gender, blood alcohol concentration, presence of disruptive behavior in the ED).

## Conclusions

Young patients admitted for alcohol intoxication are likely to develop substance misuse, mental health disorders, and social problems, suggesting they should be offered secondary prevention measures.

